# Financial incentives for smoking cessation in pregnancy: protocol for a single arm intervention study

**DOI:** 10.1186/1471-2393-13-66

**Published:** 2013-03-15

**Authors:** Theresa M Marteau, Josephine Thorne, Paul Aveyard, Julie Hirst, Rachel Sokal

**Affiliations:** 1 Health Psychology Section, King’s College London, London, UK; 2 Primary Care Clinical Sciences, University of Birmingham, Birmingham, UK; 3 NHS Derbyshire County, Newholme Hospital, Bakewell, UK; 4 NHS Derbyshire County, Scarsdale, Chesterfield, UK

**Keywords:** Smoking cessation, Financial incentives, Pregnancy, Vouchers

## Abstract

**Background:**

Smoking during pregnancy and in the postnatal period is a major cause of low birth weight and a range of adverse infant health outcomes. Stop smoking services can double quit rates, but only 17% of pregnant women smoking at the time they book for antenatal care use these services. In a recent Cochrane review on the effectiveness of smoking cessation interventions in pregnancy, financial incentives were found to be the single most effective intervention. We describe a single arm intervention study offering participation in a financial incentive scheme for smoking cessation to all pregnant smokers receiving antenatal care in one area in England. The aim of the study is to assess the potential effectiveness of using financial incentives to achieve smoking cessation in pregnant women who smoke, to inform the use of financial incentive schemes in routine clinical practice as well as the interpretation of existing trials and the design of future studies.

**Method/design:**

500 consecutive pregnant smokers are offered participation in the scheme, which involves attending for up to 32 assessments until six months post-partum, to verify smoking cessation by self report and a negative exhaled carbon monoxide measurement. At each visit when cessation is verified, participants receive a shopping voucher starting at a value of £8 and increasing by £1 at each consecutive successful visit. Assessments decline in frequency, occurring most frequently during the first two weeks after quitting and the first two weeks after delivery. The maximum cumulative total that can be earned through the scheme is £752.

**Discussion:**

The results of this study will inform the use of financial incentive schemes in routine clinical practice as well as the interpretation of existing trials and the design of future studies. The main results are (a) an estimate of the proportion of pregnant smokers who enrol in the scheme; (b) estimates of the proportion of pregnant smokers who participate in the scheme and who achieve prolonged abstinence at: i. delivery and ii. six months postpartum; (c) predictors of i. participation in the scheme, and ii. smoking cessation; and (d) estimates of the adverse effects of using incentives to achieve quitting as indexed by: i. the delay in quitting smoking to enrol in an incentive scheme and, ii. false reporting of smoking status, either to gain entry into the scheme or to gain an incentive.

## Background

### Smoking in pregnancy

Smoking during pregnancy and in the postnatal period harms the baby and mother. It is a major determinant of low birth weight and a range of adverse infant health outcomes including death [1]. Smoking in pregnancy is heavily patterned by social and material deprivation as well as age. In the United Kingdom of Great Britain and Northern Ireland (UK), women in routine and manual occupations are five times more likely than those in managerial and professional occupations to have smoked throughout pregnancy (20% vs. 4%, respectively), and 35% of pregnant women aged 20 years or younger smoked throughout pregnancy, compared with 6% of women aged 35 years and older [2]. In the US, women with fewer than 12 years education are almost four times more likely than women with more than 12 years education to smoke in the last three months of pregnancy (22.5% vs. 6.5%, respectively), with 18.5% of women aged under 20 years smoking in the last three months of pregnancy compared with 7% of those aged over 35 years [3]. These patterns of smoking contribute not only to the health inequalities associated with adult smoking but also to those in infancy following exposure to cigarette smoke before and after birth.

### Smoking cessation interventions in pregnant women

The UK has a strong tobacco control climate so cessation in pregnancy might be expected to be as high in the UK as anywhere [4]. In 2010, 26% of women in the UK smoked immediately before or during their pregnancy, with around 54% of these stopping before delivery. It is estimated that 12% [2] or 13% [5] of women smoke throughout pregnancy in the UK. Similar rates are reported in the United States of America (USA), with 12.8% of women estimated to smoke in the last three months of pregnancy, ranging from 5.1% in Utah to 28.7% in West Virginia [3]. The National Health Service (NHS) in the UK offers a free smoking cessation service to all those who want to stop smoking and promotes this to smokers. NHS support concentrates on helping women who are still smoking by the time they book for pregnancy care, usually around eight to 12 weeks of gestation. Such support typically includes approximately seven individual support sessions held in a nearby clinic over a four week period plus nicotine replacement therapies. This support has been shown to double cessation rates, but only 17% of all women smoking at booking take up the offer of support [6]. 46% of these women stop in the short-term [6], but the NHS does not record the proportion that stay abstinent throughout pregnancy.

Although some women may attempt to quit smoking and stay quit prior to and during pregnancy, relapse rates in the first 6 months after delivery are estimated at 70% [7]. Post-natal smoking and the exposure of children to environmental tobacco smoke has been linked with sudden infant death syndrome, respiratory diseases and ear infections [8]. A review of current interventions targeting relapse prevention in the post-partum period found them ineffective and recommended the evaluation of the use of financial incentives as an intervention strategy for maintaining abstinence during this time [9].

To improve long-term smoking cessation in pregnant women interventions are needed that both increase referrals to smoking services and improve long-term quit rates particularly for the vast majority of pregnant women who do not use these services.

In a recent Cochrane review on the effectiveness of smoking cessation interventions in pregnancy, financial incentives were found to be the single most effective intervention [10]. A further meta-analysis of the three most robust trials confirmed this, with women offered financial incentives having a greater chance of quitting [11]. These three trials were conducted in the USA and included only 350 women in total, leading the authors of this latter meta-analysis to recommend replication elsewhere in more robust designs using larger samples and standardised assessments of continuous abstinence.

While showing large effect sizes, the majority of women still fail to quit in these incentive schemes [12-14]. It is therefore instructive to consider the individual characteristics of those who succeed and those who fail as a basis for strengthening future schemes. One predictor of smoking cessation in pregnancy is time orientation [15]. Given a choice, people often prefer to receive smaller, more immediate rewards over larger, later ones [16]. This preference is termed delay discounting. The more people are willing to forego in order to reduce the delay for receiving a reward, the higher their delay discounting. Higher delay discounting is associated with a number of demographic characteristics including younger age, lower education and lower income [17]. Delay discounting may interact with incentive schemes to predict outcomes such that programmes offering frequent immediate rewards, as opposed to larger less frequent ones, may be particularly effective for those who discount more steeply. In one small study, the extent to which women discounted the future for themselves predicted quitting during pregnancy, with women who sustained a quit attempt beyond pregnancy discounting the future less than those who relapsed to smoking [15]. In pregnancy women may also discount the future for their babies as well as for themselves given that health decisions that a woman makes in pregnancy will affect both herself and her baby. However, it is unknown how pregnant women discount the future for themselves relative to that of their future children and whether these decisions differentially predict smoking cessation. This will be assessed in the current study.

### Unintended perverse effects of incentive schemes

There are at least two possible unintended perverse effects of incentivising smoking cessation. First, it may lead people to delay initiating a quit attempt if there is a time interval between people being informed of a scheme and their enrolment onto it. This is a particular concern in pregnancy when incentive schemes may not operate until booking for maternity care, which happens near the end of the first trimester when the majority of fetal development has already occurred. No previous study has examined this. Second, incentive schemes may encourage “gaming”, that is, people cheating a system to receive an incentive. This may occur by non-smokers falsely reporting themselves to be smokers to get onto a scheme and, amongst those on a scheme, smokers falsely reporting themselves to be non-smokers.

Few studies have investigated the precise nature or prevalence of these types of gaming. A recent review examining the use of incentives in smoking cessation studies reports limited and mixed evidence for gaming [18]. One study included in the review reported a greater discrepancy between self-reported abstinence and carbon monoxide (CO) levels for participants who were incentivized for self-reported abstinence compared to those incentivised for low CO levels. This suggests that gaming may be more likely if the incentive is not contingent on biochemical verification [19]. Another study reported that those incentivised for smoking cessation were more likely to be classified as abstinent (38%) as assessed by CO levels, compared with those not incentivized (5%), a difference that disappeared when cotinine, a more stringent test, was used to verify abstinence (7% vs. 2%, incentivised group and control group respectively) [20]. The results of this study suggest that failure to include cotinine measurement to verify abstinence will overestimate the effectiveness of financial incentives in achieving sustained quitting. More evidence regarding the nature and scale of gaming in the context of incentivizing smoking cessation is needed. In addition to informing the interpretation of incentive scheme evaluations, estimating the nature and scale of gaming in the proposed study will inform the design of incentive schemes and their evaluation.

### Current UK incentive schemes

An internet search [conducted on Monday 19^th^ November 2012, see Table 1] revealed two active UK-based schemes (one delivered as part of a routine service, and one delivered as part of a randomised controlled trial) and four recent but currently inactive UK-based schemes in which pregnant women are offered financial incentives. The incentives comprised generic shopping vouchers (n = 3), grocery vouchers (n = 1) and pharmacy vouchers (n = 1). None offered cash. The value of the vouchers ranged from £10 to £20 for each negative test result for smoking, based on carbon monoxide readings. The schemes varied in the frequency with which tests were conducted, the total amount on offer (ranging from £100 to £650), and duration, ranging from the end of pregnancy to a year after enrolment.

**Table 1:** Internet search findings for UK incentive schemes for smoking cessation in pregnancy as of 19th November 2012

**Scheme name**	**Location**	**Status**	**Country**	**Funding**	**Incentive details**	**Smoking verification method**	**URL as source**
Give it up for baby	Dundee	Active	UK	NHS	£12.50 grocery vouchers per week over 1 year	CO testing	http://www.gwumc.edu/sphhs/departments/pch/phcm/casesjournal/volume3/showcase/cases_3_09.pdf
							Accessed 19 November 2012
							http://www.nsmcentre.org.uk/sites/default/files/Give%20It%20Up%20For%20Baby%20FULL%20case%20study.pdf Accessed 19 November 2012
Healthy incentive schemes	Birmingham	Completed	UK	NHS	Up to £140 shopping vouchers over 1 year.	CO testing	http://www.stopsmokinglive.org/2010/posters/dave_jones.html Accessed 19 November 2012
Voucher Incentive Scheme	Yeovil & Torbay District Hospitals & Gloucester Royal Hospital	Completed	UK	NHS	Up to £200 in pharmacy vouchers over 1 year.	CO testing	http://www.uknscc.org/2010_UKNSCC/presentations/elaine_watson.html - Accessed 19 November 2012
Significant Other Supporters (SOS)	Yorkshire and Humber Piloted in 12 stop-smoking services	Completed	UK	NHS	Up to £200 in shopping vouchers over 1 year	CO testing	http://smoking-quit.info/mothers-paid-to-quit-cigarettes Accessed 19 November 2012
	The North East Essex (Colchester)	Completed	UK	NHS	Up to £100 grocery vouchers over 1 year	CO testing	http://www.uk.coop/pressrelease/food-vouchers-incentive-pregnant-smokers - Accessed 18 March 2013
The Cessation in Pregnancy Incentives Trial (CPIT)	Greater Glasgow and Clyde	Active	UK	Chief Scientist Office Scottish Government (sponsored by the NHS)	Up to £400 in shopping vouchers for abstinence up to 34 to 38 weeks gestation	CO testing and urine test (for final payment)	http://www.controlled-trials.com/ISRCTN87508788/ - Accessed 19 November 2012
							Trial protocol available at http://www.trialsjournal.com/content/13/1/113 Accessed 19 November 2012
HERS (Health Enhancement Reward scheme)	West Suffolk (Bury St Edmunds and Sudbury)	Completed	UK	NHS	Up to £120 in vouchers (£10 per month) for baby products, beauty treatments and cinema tickets.	CO testing	Health Enhancement Reward Scheme (HERS) Project Evaluation - UCS Full Report January 2010
							http://www.suffolk.nhs.uk/Home/Publications/20092010.aspx Accessed 19 November 2012

From information available on websites and from our knowledge of financial incentives schemes currently running in the UK, only one is part of a randomised trial (the Cessation in Pregnancy and Incentives Trial [21]). The current study differs from this trial by using a more frequent incentive schedule (a maximum of 32 incentive points compared with four), offered for longer (up to six months postpartum compared with 36 weeks gestation), and in which the size of incentives is incremental, as opposed to fixed. The incentive schedule for the current study (see Table 2) is based on those used in two USA studies which reported large effect sizes [13,14].

**Table 2:** **Incentive schedule**^**1**^

**Visit no.**	**Visit timepoint**	**Incentive (£)**	**Cumulative total (£)**
1	1 day after quitting	8	8
2	3 days after quitting	8 + 1	17
3	7 days after quitting	9 + 1	27
4	12 days after quitting	10 + 1	38
5	3 weeks after quitting	11 + 1	50
6	4 weeks after quitting	12 + 1	63
7	5 weeks after quitting	13 + 1	77
8	6 weeks after quitting	14 + 1	92
9	8 weeks after quitting	15 + 1	108
10	10 weeks after quitting	16 + 1	125
11	12 weeks after quitting	17 + 1	143
12	14 weeks after quitting	18 + 1	162
13	16 weeks after quitting	19 + 1	182
14	20 weeks after quitting	20 + 1	203
15	24 weeks after quitting	21 + 1	225
16	28 weeks after quitting	22 + 1	248
DELIVERY			
17	2 day after delivery	23 + 1	272
18	4 days after delivery	24 + 1	297
19	7 days after delivery	25 + 1	323
20	12 days after delivery	26 + 1	350
21	3 weeks after delivery	27 + 1	378
22	4 weeks after delivery	28 + 1	407
23	5 weeks after delivery	29 + 1	437
24	6 weeks after delivery	30 + 1	468
25	8 weeks after delivery	31 + 1	500
26	10 weeks after delivery	32 + 1	533
27	12 weeks after delivery	33 + 1	567
28	14 weeks after delivery	34 + 1	602
29	16 weeks after delivery	35 + 1	638
30	20 weeks after delivery	36 + 1	675
31	24 weeks after delivery	37 + 1	713
32	28 weeks after delivery	38 + 1	752

^1^ This table shows the maximum that can be earned by a woman who starts on the scheme at about 12 weeks gestation and who attends all scheduled visits and is verified as not smoking at each visit. A woman starting quitting later in pregnancy would start with Visit 1 in terms of the incentive on offer. If she delivered shortly after Visit 12, her post delivery visit is paid at the rate of Visit 13. The maximum number of visits this woman could make is 28.

### Public attitudes

The use of financial incentives attracts controversy. A recent analysis of media coverage revealed that most articles covered a mix of views [22]. Preliminary results from a series of ongoing experiments reveal that the majority of participants were prepared to trade-off their negative attitudes towards financial incentives against increased effectiveness, i.e. the more effective a scheme, the more acceptable it is judged to be [23].

We describe here a single arm intervention study to be conducted within NHS Derbyshire County at the Chesterfield Royal Hospital NHS Foundation Trust. Between April 2010 to March 2011, 2881 women received care at Chesterfield Royal Hospital NHS Foundation Trust at delivery, out of which a similar proportion smoked at booking (Women smoking at booking n = 500, 17% of total women booking) as they did at delivery (Women smoking at delivery n = 483, 17% of total women delivering). The current study will describe smoking cessation rates in all smokers receiving antenatal care from midwives based at one hospital, Chesterfield Royal Hospital NHS Foundation Trust, for a 12 month period. During this period all pregnant women who smoke will be offered participation in a financial incentive scheme for smoking cessation, regardless of whether they are interested in stopping smoking or planning to use stop smoking services. The current study can therefore be characterized as a “cessation induction” study, as distinct from an “aid to cessation” study, meaning that it aims to encourage women who would not have tried to quit smoking to do so.

## Study aim and objectives

The aim of this study is to assess the potential effectiveness of using financial incentives to achieve smoking cessation in pregnant women who smoke, to inform the use of financial incentive schemes in routine clinical practice as well as the interpretation of existing trials and the design of future studies.

The specific objectives of the study are:

1) To estimate the proportion of pregnant women who smoke at the point of receiving antenatal care from midwives, at one hospital over a 12 month period, who accept the offer to participate in a financial incentive scheme for smoking cessation

2) To estimate the proportion of pregnant women who smoke, and who participate in the scheme and achieve prolonged abstinence at (a) delivery and (b) six months post-partum

3) To examine predictors of (a) participation in the scheme, and (b) smoking cessation

4) To estimate the prevalence of two possible sets of adverse effects of using incentives to achieve quitting:

a) Delay in quitting, in order to enrol in an incentive scheme

b) Gaming *i.e.* false reporting of smoking status:

i) to gain entry to the scheme (i.e. non-smokers acting as smokers)

ii) to gain an incentive (i.e. smokers acting as non-smokers)

5) To describe the process of visits for incentive payment assessments both in patients’ homes and in clinic settings. This will include the total number of visits, the length of each visit, visits defaulted, mileage travelled and total time taken.

## Methods/Design

The study has been approved by the Derbyshire Research Ethics committee [Ref no.11/H0401/2].

### Overview of design

This is a single arm intervention study in which pregnant smokers attending for antenatal care are offered participation in a financial incentive scheme for smoking cessation.

### The incentive scheme

Pregnant women participating in the scheme will be asked to provide verification of smoking cessation on up to 32 occasions, depending on when they enrol (see Table 2). Women must declare complete abstinence since last contact, validated by a “test-negative” exhaled carbon monoxide (CO) measurement, i.e. a concentration of less than or equal to 6 ppm. At each visit when cessation is reported and validated, participants will receive a voucher for shopping items. The value of the voucher received at the first visit will be £8, increasing by £1 at each consecutive visit at which smoking cessation is reported and validated using CO measurement.

The scheme has the following four characteristics:

*Type*: the incentive offered is a voucher card which can be topped-up and used to redeem a range of goods including groceries, clothes, baby and other consumer goods. It cannot be used in exchange for cigarettes or alcohol.

*Magnitude*: the total amounts offered are £248 for abstinence during pregnancy and £504 for abstinence six months post partum. These are similar to the amounts offered in the three randomised controlled trials conducted in the US, each of which reported large effects on abstinence [12-14]. The whole sum is available only to women who sustain abstinence and verify this biochemically for the entire duration of this period.

*Duration*: each participant in the incentive scheme can be enrolled for around a year, to maximise abstinence during pregnancy and in the first six months post-partum.

*Frequency*: incentives are offered more frequently during the first two weeks of quit attempts to maximise quitting in this time period, which is a major predictor of ultimate success [24]. Increasing the amount that can be earned with abstinence is shown in laboratory studies to be more effective than offering constant or decreasing amounts [25]. Once a behaviour is established, reducing the frequency of rewards is important in maintaining the change [26]. Our proposed incentive structure reflects these three sets of observations (Table 2). Two of the three US trials used this structure [13,14]. None of the UK pilot schemes of which we are aware has used this structure.

The incentive scheme ‘starts again’ after birth for two reasons. First, women who have smoked during pregnancy may now wish to become abstinent to avoid affecting their child’s health through environmental tobacco smoke and/or breast milk. For them, this incentive frequency mirrors that during pregnancy for the initial days of a quit attempt. Second, we know that return to smoking after pregnancy is a deliberate choice by many mothers when the incentive to be abstinent due to carrying a baby attached by a placenta is removed. The incentive structure is designed to reward a woman frequently during the initial postpartum period to maintain the incentive to be abstinent when the natural incentive to do so has gone.

### Measuring smoking cessation

To assess the effectiveness of the intervention it is necessary to have an accurate measure of smoking cessation. For most people who smoke cigarettes and have difficulty quitting (addicted smokers), only the state of complete abstinence is sustainable as occasional smoking leads to reinstatement of fulltime smoking. The outcome of choice for smoking cessation studies should therefore be prolonged or continuous abstinence [27,28]. In particular, this is the only outcome known to be associated with health benefits.

Biochemical verification tests, such as testing CO levels or nicotine metabolites such as cotinine, are the preferred methods of validating smoking cessation in clinical studies such as this [29]. Testing for CO levels using a breath test assesses smoking in the preceding one to four hours whereas cotinine analysis from blood, urine or saliva samples can measure exposure to smoking in the preceding few days [29]. However, testing for cotinine in body fluids is more complex to administer and expensive to analyse than testing CO levels.

In the current study we will use cotinine from urine samples to confirm smoking status at recruitment, and from saliva samples to verify abstinence both at delivery and at six months post-partum. Cotinine testing will also be used once during pregnancy (at around 28 weeks) to compare with CO measurements taken at the same time. For practical reasons, CO tests alone will be used on all other occasions when women attend to receive incentive payments.

### Study outcomes

#### Smoking cessation: assessed on two occasions

Self-reported, prolonged complete abstinence from smoking (a) between six weeks after enrolment in the scheme and prior to childbirth; and (b) between childbirth and six months later. All women lost to follow-up will be assumed to have resumed smoking. This definition gives women a cessation induction period of up to six weeks, although we expect many women will establish abstinence sooner than this.

These are two outcomes requiring the following different measures:

(a) between six weeks after enrolment in the scheme and prior to childbirth

i. self reported smoking cessation for at least 24 hours at six weeks after enrolment in the scheme, validated by exhaled CO measurement of less than or equal to 6 ppm;

ii. self reported complete abstinence from 6 weeks after enrolment to 36 weeks gestation, validated by salivary cotinine less than 15 ng/ml at 36 weeks. In addition, there must be no reported lapses and a record that all CO readings taken between these dates indicate non-smoking status. All women lost to follow-up will be assumed to have resumed smoking.

(b) between childbirth and six months later

i. self reported smoking cessation for at least 24 hours two days after delivery, validated by exhaled CO measurement of less than or equal to 6 ppm and salivary cotinine less than 15 ng/ml, in keeping with local practice.

ii. self reported complete abstinence from two days after enrolment to six months postpartum, validated by salivary cotinine less than 15 ng/ml at six months. In addition, there must be no reported lapses and a record that all CO readings taken between these dates indicate non-smoking status [27]. All women lost to follow-up will be assumed to have resumed smoking.

##### Other measures of smoking status

Self reported prolonged abstinence from smoking assessed by self-reported smoking cessation six weeks after enrolment in the scheme, validated by CO reading, and self report smoking cessation at 28 weeks gestation, validated by salivary cotinine less than 15 ng/ml.

##### Participation in the scheme

This will be recorded by the study support worker and presented as a proportion of all women routinely recorded at booking consultations as smoking.

##### Predictors of smoking cessation

Women will complete a questionnaire when they are recruited to the scheme to assess the following: delay discounting for self and the baby, using an adapted version of Kirby and Marakovic’s task [16]; nicotine addiction, using the Fagerstrom test for nicotine dependence [30]; and, socio-economic status, assessed using postcode as an index of area level deprivation.

#### Estimating the adverse effects of using incentives to achieve quitting

a) *delay in quitting smoking to enrol in an incentive scheme*: There are concerns that the estimated 11% of women who quit smoking upon learning they are pregnant may delay quitting until they can enrol in the incentive scheme. We will estimate this effect by comparing the self-reported smoking status of women during the time the scheme is running, with that recorded for the preceding 12 months. While this is open to the usual biases associated with historic controls, it will provide some indication of whether the scheme has delayed quitting.

b) *gaming i.e. false reporting of smoking status:* Two types of gaming are possible;

i) *To gain entry to the scheme (i.e. non-smokers acting as smokers).* We will estimate this in two ways: first, using historic controls (by comparing the proportion of women who claim to smoke at the outset of the study with the proportion who reported being smokers in the year prior to the start of the scheme); second, by assessing the proportion of women claiming to smoke with levels of urinary cotinine compatible with non-smoker status. Our inclusion criteria are set to minimise entry to the scheme by non-smokers.

ii) *To gain an incentive (i.e. smokers acting as non-smokers).* We will estimate this by validating CO records and self-reported smoking status against measurement of cotinine collected at 28 weeks gestation, 36 weeks gestation and 2 days after delivery and 6 months post-partum.

The two primary outcomes of this study are:

smoking cessation at delivery; and,

false reporting of smoking status to gain an incentive.

The remaining outcomes will be considered secondary.

#### Fidelity to protocol

Fidelity to protocol checks will comprise checking recorded CO measurements against incentives paid, and dates on which visits were scheduled and dates on which these occurred.

#### Barriers and facilitators

Two interviews will be conducted via telephone with all women recruited to the scheme six weeks after recruitment and four weeks after their participation in the scheme ends. The aims of these interviews are to explore barriers and facilitators to success.

### Participant selection and withdrawal

All women reporting smoking and who are booked for antenatal care by midwives employed by Chesterfield Royal Hospital NHS Foundation Trust, over a 12 month period, will be informed about the scheme regardless of where they choose to deliver.

#### Inclusion criteria

Eligible pregnant women are those who meet the following smoking criteria:

▪ Pregnant women who self report as smokers

▪ Pregnant women who have a urinary cotinine concentration of 1.5 mg/ml or above

#### Exclusion criteria

▪ Women who are unable to provide informed consent.

▪ Women who are under 16.

▪ Women who do not speak English.

Women who drop out from the scheme will be counted as smokers in any analysis unless they have died or moved to an untraceable address in which case they will be excluded from the numerator and the denominator, in line with Russell standard criteria for reporting smoking cessation outcomes from studies [27].

Women who miscarry or have a stillbirth will be allowed to continue in the scheme if they so choose. They will, however, be excluded from the numerator and denominator of the cessation rate, in line with Russell standard criteria [27]. For these women the date of miscarriage or stillbirth will be classed as the date of delivery and the incentive schedule adjusted accordingly.

### Recruitment and screening

Midwives currently record the smoking status of all women they book for antenatal care. All women reporting to a midwife at booking that they smoke occasionally or every day will be referred to the Derbyshire Community Healthcare Service (DCHS) Stop Smoking Service (SSS) as is current standard care. Forms describing the smoking status of all women being booked during the pilot period will be posted first class to the SSS. The midwife will give women who smoke two leaflets: one that outlines the incentive scheme and one outlining the stop smoking services.

Women who smoke will be phoned by a support worker from the SSS within one working day of receipt of their referral from the midwife. The support on offer will be described, namely stop smoking services and the financial incentive scheme. Women will be informed that the latter is for heavier smokers with joining dependent upon a urinary cotinine concentration above a certain level (at least 1.5 mg/ml).

### Enrolment

**Figure 1:**
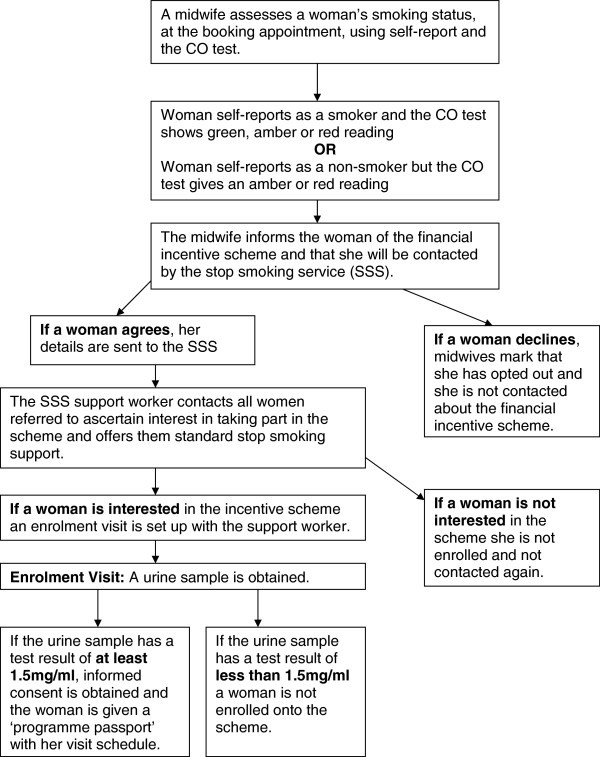
Flow of recruitment process.

Women wanting to be considered for the scheme will be posted the participant information sheet and a home visit with the support worker will be arranged. At this enrolment visit a urine sample will be tested to confirm eligibility for the scheme, using a point of care test. (See Figure 1 for recruitment process).

All women will be offered the standard support by the SSS regardless of whether they take part in the financial incentive scheme.

Pregnant women who meet the inclusion criteria will be enrolled into the study. Provided they have read and understood the participant information sheet and had the chance to have any questions answered, signed consent will be sought for the following:

(a) participation in the scheme;

(b) collection and analysis of one urine and four saliva samples for cotinine estimation: two during pregnancy (28 and 36 weeks gestation) and two post-partum (two days after delivery, and six months post-partum);

(c) completion of a questionnaire to assess predictors of smoking cessation.

Once consent has been given, the support worker will encourage the participant to set a quit date and issue them with a ‘programme passport’ which will contain their photograph for identification and the date, test result and voucher value of each visit.

### Starting a quit attempt

After this visit, once a woman has set a quit date, she contacts the support worker by phone to arrange for the support worker to visit her at home, within 24 hours.

### Visits

These visits are conducted by the support worker who records all visits manually on client record cards; this information is subsequently entered into the study database.

During pregnancy, CO tests will take place on up to 16 occasions (depending upon when in pregnancy women enrol in the scheme), most frequently in the first two weeks (4 occasions) with decreasing frequency until delivery. After delivery and until 6 months postpartum, testing will also take place on 16 occasions; with decreasing frequency (Table 2). If participants are not available on the scheduled visit date or the date falls on a weekend, the visit will be rescheduled for one working day before or after the scheduled date for the first 1–4 visits and two working days for the following visits.

At the first visit participants are given a voucher card and receive £8 on it for a negative CO test. The sum given at each visit increases by an increment of £1 for every successive negative CO test.

Failure to attend for a scheduled visit or testing positive for smoking means no incentive is given at that visit and the incentive value is reset to the baseline of £8 at the next successful visit. Following two consecutive successful test results, the incentive value is re-set to the highest point attained prior to the positive for smoking test result.

Women can continue in the scheme for as long as they choose but incentives are only provided to those stopping smoking.

CO testing is undertaken in a woman’s home for the first four weeks and thereafter women are required to attend one of five drop-in clinics held in the local community. If a participant cannot get to a drop-in session for a visit due to illness or the visit is too close to delivery, the support worker visits the woman at home.

At each visit, whether at a home or at a community clinic, the support worker does the following:

1. checks and records smoking status: self report and CO measurement

2. if the woman reports she is not smoking, and her CO reading is ≤6 ppm, then:

a. voucher payment is approved

b. the results, date of the next visit and the value of the next incentive payment are recorded in the woman’s hand held notes.

3. if the woman reports she is smoking or her CO reading is ≥6 ppm, then

a. voucher payment is not approved

b. the procedure for the next visit is discussed

c. the results, date of next visit and the value of the next incentive payment (£8) is recorded in her hand held notes.

4. the support worker answers any questions and provides general encouragement; women wanting smoking cessation advice are encouraged to use the NHS stop smoking service.

The duration of each visit is between ten and 25 minutes.

#### Missed visits

If a woman is not at home when a scheduled visit was arranged, the support worker calls the woman from the latter’s doorstep. If no response is obtained, a prepared note is left at the woman’s home requesting she contacts the support worker in the next 48 hours. If no contact is made, the support worker contacts the woman. A similar procedure is followed for women who do not attend an appointment scheduled in the community, with follow up letters posted, rather than being left at her home.

### Biological samples taken to assess quitting and gaming

A urine sample is tested at the enrolment visit, using a dipstick test, to assess eligibility for the scheme, with urinary cotinine concentrations equal to or more than 1.5 mg/ml sufficient for enrolment.

Saliva samples are collected by the support worker at 28 weeks gestation, 36 weeks gestation, 2 days post delivery and amongst women reporting cessation, at 6 months post delivery and analysed by a nationally accredited laboratory for cotinine concentrations. For women reporting current use of nicotine replacement therapy, anabasine, a tobacco specific alkaloid, will be analysed.

In addition to being used to biochemically verify quitting, the results of these analyses will be used to estimate the proportions of women reporting having quit who have not. Both women and the support worker will remain blinded to the test results unless a woman requests her result. Women proven to be smoking on any of these extra tests (i.e. those using saliva as opposed to CO) will not be confronted with this, withdrawn from the scheme or refused vouchers. The aim is to estimate the extent of the gaming that is occurring. It is possible for women to abstain for a few hours and pass a CO test but such women would be shown to be smokers by salivary cotinine.

### Post-recruitment retention

Women enrolled in the scheme who do not keep pre-arranged meetings to report their smoking status as per the scheme protocol are contacted by telephone by a support worker. Failure to respond after three attempts at contact is assumed to signal withdrawal from the scheme.

### Data collection and management

Data on women’s progress through the scheme are collected by the support workers and entered onto a data base by a member of the evaluation team. Data on biochemical tests are entered by the evaluation team with the support worker remaining blinded to these results.

### Economic evaluation

No formal economic evaluation is planned for this study. The total budget set aside over two years for the scheme by the Primary Care Trust is £139,000. These costs cover the employment of 1.5 support workers (paid on a pay scale in the range £16,110-£19,077), the costs of the incentives and the laboratory costs for saliva testing. This money does not cover the costs of NHS Stop Smoking Advisors who women access through the existing scheme.

### Precision estimation

This is a single arm intervention study that aims to estimate the proportions of women who join the scheme and who achieve prolonged abstinence, as well as the proportion of women who falsify smoking status to gain rewards. We will offer the scheme for 12 months which will likely involve a group of an estimated 500 pregnant women based upon the number of smokers booking for care at Chesterfield Royal Hospital NHS Foundation Trust in the preceding 12 month period. We estimate that about 30% of smokers approached will enrol, based on 45% recruitment rate in a “cessation induction” trial of incentives that used similar eligibility criteria, conducted in the US [14], and estimated 14% and 10% recruitment rates to two recent UK “aid to cessation” trials for pregnant women trying to quit smoking and not involving any financial incentives [31,32]. A population of 500 will provide an estimate in the region of 30% recruitment with a precision of +/− 4%, using 95% confidence intervals. Estimating 24% quit rates in the 150 enrolled women, based on estimates generated in a recent systematic review [10] will provide an estimate with a precision of +/− 7%, using 95% confidence intervals. We conservatively estimate that the proportion of women quitting will be larger than zero, the rate observed for the preceding period of observation in the study hospital. All women lost to follow-up will be assumed to have resumed to smoking. It is planned to recruit all smokers, regardless of motivation to stop smoking.

### Data analysis

Outcomes will be reported as proportions with 95% confidence intervals for the following: (a) the proportion of pregnant smokers who enrol in the scheme; (b) the proportion of pregnant smokers who participate in the scheme and who achieve prolonged abstinence at i. delivery and ii. six months postpartum; and, (c) the proportion of women who falsely report smoking status to gain entry into the scheme and to gain an incentive. No formal statistical tests will be performed. Rates of prolonged abstinence will be considered to suggest a positive impact of the scheme if the 95% confidence intervals do not include 0%. This is the rate of cessation recorded in a similar population of women in the preceding 12 months in the hospital where the study is taking place.

### Reporting of study results

The results of this study will be submitted for publication in a peer reviewed journal in December 2013, three months after the last data point will have been collected.

## Discussion

Two aspects of the design of this study limit the inferences that can be made regarding the impact of the incentive scheme on quitting. First, the study does not include a comparison group that receives no intervention. Second, the intervention can be considered a complex behavioural intervention involving not only the provision of incentives but support that is additional to standard care, a limitation that is evident in most, if not all, studies of incentive schemes [33]. The study will nonetheless provide estimates of the participation and drop-out rates for the scheme and an indication of possible effect size. It will also provide rigorous data on “gaming” in incentive schemes, in a way that no other study has so far done. These latter data have implications for the interpretation of previous studies as well as the design of subsequent studies using financial incentive to motivate smoking cessation.

## Competing interests

Paul Aveyard has done consultancy and/or research for manufacturers of smoking cessation medication.

## Authors’ contributions

TM, JT, PA, JH and RS all helped to develop the design of the research study. TM drafted the protocol and advised on the use of financial incentives. PA provided expertise on smoking cessation in pregnancy and the use of biochemical measures. All authors have read and approved the final manuscript.

## Pre-publication history

The pre-publication history for this paper can be accessed here:

http://www.biomedcentral.com/1471-2393/13/66/prepub
